# With professional overexposure, how to protect yourself from litigation?

**DOI:** 10.1590/2177-6709.23.4.007-008.edt

**Published:** 2018

**Authors:** Flávia Artese

**Affiliations:** 1 Universidade do Estado do Rio de Janeiro, Departamento de Odontologia Preventiva e Comunitária (Rio de Janeiro/RJ, Brazil). Universidade do Estado do Rio de Janeiro Universidade do Estado do Rio de Janeiro Departamento de Odontologia Preventiva e Comunitária Rio de JaneiroRJ Brazil

The last decades have witnessed important and profound changes in communications. We exchanged long waits for desired telephone calls, to instant text messages via apps, where we can even acknowledge if the subject is online or not. The world becomes small, reachable at our fingertips by means of portable devices, and the information becomes horizontal, blurring social differences. This generates knowledge and at the same time the demand for services.

It is quite natural that the healthcare sector also uses digital communication to offer its services, following the trend of other areas. For Orthodontics, the most recent data that we know of is a paper published by Lindauer and collaborators that evaluated the preferences of orthodontists and patients in relation to social media. The results demonstrated that, in the United States, 76% of orthodontic offices used social network, of which Facebook was the most common, and 59% had a website.^1^

Even though the Internet brought many positive aspects to dental practice, fraudulent and unethical advertisement are growing, as for example, false advertising^2^ and those that disobey the professional principles of ethics and code of conduct. In Brazil, we do not have such data, but due to the most recent negative happenings, the professional overexposure in social media is hitting the headlines of the main newspapers in the country.^3^ This has to be analyzed, since tools that should be used to help in professional marketing can, in reality, because of bad use, tarnish the professional image.

In the Special Article of this issue of the *Dental Press Journal of Orthodontics* (pages 88 to 93), a group of researchers from the Federal University of Juiz de Fora evaluated the risks that orthodontists have to be involved in litigation with indemnity purposes. They concluded that there is significant lack of knowledge on the part of professionals regarding the specific obligations and duties as providers, objective or subjective responsibility, character of the professional activity and the influence of the Brazilian laws protecting the consumer. They also observed that, in addition, there is a deficiency in professional training, absence of a professional contract, poor organization and storage of records and an inadequate patient follow-up during and after treatment.

This paper highlights that one of the main reasons for compensatory litigations is the failure of patient-professional trust. In general, the orthodontist does not recognize that there is an immediate contract established when the patient begins treatment, and that an indispensable instrument for his protection is the free informed consent, wherein the patient declares himself aware of what was agreed for his treatment.

On the other hand, the way orthodontic treatment is being publicized has dramatically changed. In the United States, the referral of patients to orthodontists has fallen significantly due to aggressive advertising campaigns targeted directly at the patient and to the possibility of the treatment to be performed by general dentists. The offer of short-term treatments only for esthetical correction of teeth is growing more and more.^4^

In the market scenario, it is becoming more common to offer to the patient only what he wants. And even with an informed consent, there is a very thin line between not approaching certain problems and allowing others to remain untreated, and in this way one may be practicing below the standard of care.^5^ In this case, the patient’s informed consent is useless to protect the professional, since he is the one supposed to recognize and to be responsible for the health integrity of his patient.

We recommend this Special Article so that the professional can situate himself in his needs for legal protection. It is important to state that constant professional updating is necessary so that the orthodontist can offer the best and safest level of treatment, and therefore of health.

Good readings!

Flavia Artese - editor-in-chief (flaviaartese@gmail.com)
