# Supplementary material for: With professional overexposure, how to protect yourself from litigation?
Source: Dental Press J Orthod. 2018 Jul-Aug;23(4):7–8. doi: 10.1590/2177-6709.23.4.007-008.edt (PMC6150706; doi:10.1590/2177-6709.23.4.007-008.edt)
Supplement: Translation

# Translation: Com a superdivulgação profissional, como se proteger de brigas judiciais?

**Authors:** Flávia Artese

**Affiliations:** 1 Universidade do Estado do Rio de Janeiro, Departamento de Odontologia Preventiva e Comunitária (Rio de Janeiro/RJ, Brasil).

As últimas décadas foram assoladas por importantes mudanças nos meios de comunicação. Saímos de longas esperas por aquele desejado telefonema, para mensagens instantâneas de texto, via aplicativos, onde podemos até saber se o indivíduo encontra-se “online” ou não. O mundo se torna pequeno, alcançável ao toque dos dedos por meio de aparelhos portáteis, e a informação se horizontaliza, desfazendo diferenças sociais. Isso gera conhecimento e, ao mesmo tempo, a demanda por serviços.

Nada mais natural do que a área de saúde também se utilizar das comunicações digitais para oferecer seus serviços, seguindo a tendência de outras áreas. Para a Ortodontia, os dados mais recentes que conhecemos são de um trabalho publicado em 2015 por Lindauer e colaboradores, que avaliaram as preferências de ortodontistas e pacientes com relação às mídias sociais. Os resultados demonstraram que, nos Estados Unidos, 76% dos consultórios usavam redes sociais, sendo o Facebook o mais utilizado, e 59% possuíam um website^1^. 

Apesar da internet ter trazido inúmeros aspectos positivos, mecanismos de divulgação fraudulenta e antiética estão crescendo, como, por exemplo, a propaganda enganosa^2^ e aquela que fere os códigos de ética do exercício profissional. Para o Brasil, desconhecemos esses dados; mas, devido aos recentes acontecimentos negativos, o grau de superexposição dos profissionais de saúde nas redes sociais está sendo matéria de primeira página nos principais jornais do país^3^. Uma reflexão é necessária, pois ferramentas que deveriam estar ajudando o *marketing* profissional podem, na realidade, devido ao seu mau uso, estar fragilizando juridicamente os profissionais.

No Tópico Especial desta edição do *Dental Press Journal of Orthodontics* (pgs. 88 a 93), um grupo de pesquisadores da Universidade Federal de Juiz de Fora avaliou os riscos que levam os profissionais da área de Ortodontia a serem envolvidos em disputas judiciais com fins indenizatórios. Concluíram que existe significativo desconhecimento dos profissionais quanto às suas obrigações específicas e deveres como prestadores de serviços, responsabilidade objetiva ou subjetiva, caracterização da atividade profissional (obrigação de meio ou resultado) e à influência do Código do Consumidor. Observaram, também, que, associada a isso, há uma deficiência na capacitação do profissional, ausência de relação contratual, falta de organização e manutenção de documentação ortodôntica e acompanhamento deficiente do paciente durante e após o tratamento. Esse artigo salienta, ainda, que um dos principais motivos para as lides judiciais indenizatórias é a falência das relações profissional-paciente. Em geral, o ortodontista não reconhece que há uma situação contratual tão logo o paciente inicia tratamento, e que um instrumento indispensável para sua proteção é o consentimento livre informado, onde o paciente declara-se ciente do que foi acordado para seu tratamento.

No entanto, a forma como o tratamento ortodôntico vem sendo divulgado para o público mudou radicalmente. Nos Estados Unidos, a indicação de pacientes para os especialistas em Ortodontia caiu de maneira drástica, devido a campanhas publicitárias agressivas de empresas voltadas diretamente para o paciente e à possibilidade do exercício dessa por generalistas. A oferta de tratamentos a curto prazo, com a correção apenas estética dos dentes, vem aumentando cada vez mais^4^.

Nesse cenário atual do mercado, está cada vez mais comum oferecer ao paciente apenas o que ele deseja. E, mesmo com um consentimento livre e esclarecido, existe uma linha tênue entre deixar de corrigir certas disfunções e permitir que outras se mantenham e, com isso, exercer a profissão abaixo do que é conhecido como “*standard of care*”^5^. E, nesse caso, o consentimento do paciente em nada irá proteger o profissional, pois cabe a ele reconhecer e se responsabilizar pela integridade da saúde do seu paciente. 

Recomendamos a leitura deste Tópico Especial para que o profissional possa se situar nas suas necessidades de proteção judicial. Reforçamos que a atualização profissional constante é necessária para que possa estabelecer e oferecer ao seu paciente o melhor e mais seguro nível de tratamento e, portanto, de saúde.

Boa leitura!

Flavia Artese - editora-chefe (flaviaartese@gmail.com)
